# Validation of Screening Questions for Hyperacusis in Chronic Tinnitus

**DOI:** 10.1155/2015/191479

**Published:** 2015-10-18

**Authors:** Martin Schecklmann, Astrid Lehner, Winfried Schlee, Veronika Vielsmeier, Michael Landgrebe, Berthold Langguth

**Affiliations:** ^1^Department of Psychiatry and Psychotherapy, University of Regensburg, Germany; ^2^Interdisciplinary Tinnitus Center, University of Regensburg, Regensburg, Germany; ^3^Department of Otolaryngology, University of Regensburg, Germany; ^4^Department of Psychiatry and Psychotherapy, Kbo-Lech-Mangfall-Klinik, Hausham, Germany

## Abstract

*Background.* We investigated the validity of the two hyperacusis items of the TSCHQ (Tinnitus Sample Case History Questionnaire) from the TRI (Tinnitus Research Initiative) database by comparing them with the German hyperacusis questionnaire GÜF.* Methods.* We investigated the association of the GÜF with the TSCHQ screening questions for both the sum score and the single items with correlation, contrast, principal component, and discriminant analysis in a sample of 161 patients with chronic tinnitus.* Results.* TSCHQ items and the GÜF total score were significantly associated with a special focus on fear and pain related hyperacusis. Factor analysis of the GÜF revealed the three factors “fear and pain related hyperacusis,” “hearing related problems,” and “problems in quality of life.” A discriminant analysis showed a sensitivity of 64% and a specificity of 71% of the TSCHQ items for the establishment of tinnitus patient subgroups with and without hyperacusis.* Discussion.* Both hyperacusis TSCHQ items can serve as screening questions with respect to self-reported hyperacusis in chronic tinnitus with a specific focus on fear and pain related hyperacusis. However, the multiple dimensions of hyperacusis should be considered for diagnosis and treatment in both scientific and clinical contexts.

## 1. Introduction

Hyperacusis has several definitions, but all of them include intolerance to “normal” sounds [1]. “Normal” is defined as an intensity or volume of a perceived sound that would not bother a person with “standard” hearing. Hyperacusis is described in terms of discomfort, pain, hypersensitivity, or hyperresponsiveness and can be related to the domains “loudness,” “annoyance,” “fear,” and “pain” [2]. The importance of anxiety and avoidance behavior in hyperacusis was recently corroborated [3].

Two inventories exist for the assessment of hyperacusis. The first inventory differentiates hyperacusis into the factors “cognitive reactions to hyperacusis,” “actional/somatic behavior,” and “emotional reaction to external noises” (Geräuschüberempfindlichkeitsfragebogen; GÜF: engl. hypersensitivity to sound questionnaire) [4]; the second defines it using attentional, social, and emotional dimensions (hyperacusis questionnaire; HQ) [5].

From a psychoacoustical perspective, hyperacusis is related to loudness recruitment. Loudness recruitment can usually be found in hearing loss with abnormal rapid increases of perceived loudness with increasing sound intensity [6]. There is evidence that hyperacusis and loudness recruitment are not identical [7], but the two phenomena are also not exclusive from each other [2, 6].

The analysis of a large tinnitus database [8] revealed that hyperacusis is highly prevalent among patients with chronic tinnitus and that hyperacusis characterizes a specific subtype of tinnitus patients with a greater need for treatment [9]. Thus, screening tools for hyperacusis are necessary in the diagnostic assessment of chronic tinnitus. We investigated the validity of the two hyperacusis items (“Do you have a problem tolerating sounds because they often seem much too loud? i.e., do you often find too loud or hurtful sounds which other people around you find quite comfortable?” “Do sounds cause you pain or physical discomfort?”) of the TSCHQ (Tinnitus Sample Case History Questionnaire) [10] from the TRI (Tinnitus Research Initiative) database [8], by correlating them with the German GÜF. First, we specified descriptive data of the different hyperacusis measures in a sample of 161 patients with chronic tinnitus. Second, we investigated the association of the screening items with the GÜF sum score using correlational analyses. Further fine-grained analyses included linear discriminant, principal component, and correlation analyses with tinnitus-specific and tinnitus-unspecific parameters to assess the dimensions of hyperacusis which can be measured with the screening items. These analyses were performed to assess the validity of the TSCHQ screening items as screening parameters for hyperacusis as identified with the GÜF.

## 2. Materials and Methods

The 161 subjects were patients of the Interdisciplinary Tinnitus Center at the University of Regensburg (Regensburg, Germany). Tinnitus diagnosis at the Department of Otorhinolaryngology and the Department of Psychiatry included a complete otologic and audiologic examination with pure tone audiometry, tympanometry, and otoscopy. Patients gave written informed consent for their data to be used in the Tinnitus Research Initiative database which was approved by the Ethics Committee of the University Hospital of Regensburg (Germany; reference number 08/046).

Patients completed the tinnitus questionnaire (TQ; range: 0–84) [11, 12], the Tinnitus Handicap Inventory (range: 0–100) [13], five numeric rating scales for the assessment of tinnitus loudness, annoyance, discomfort, ignorability, and unpleasantness (scale: 0–10), the Geräuschüberempfindlichkeitsfragebogen (GÜF; engl. hypersensitivity to sound questionnaire; range 0–45) [4], a German version of a quality of life scale (WHOQOL-BREF; scores 4–20) [14], and the Beck depression inventory (BDI; range: 0–63) [15]. In the quality of life scale high scores indicate high quality of life; in all other scales high scores indicate high burden. The TQ contains different subscales: emotional distress, cognitive distress, sleep disturbance, auditory perceptual difficulties, somatic complaints, and intrusiveness.

We used the two screening questions for hyperacusis as indicated by the Tinnitus Sample Case History Questionnaire [10]: (1) “Do you have a problem tolerating sounds because they often seem much too loud? That is, do you often find too loud or hurtful sounds which other people around you find quite comfortable?” with the answers “never, rarely, sometimes, usually, or always” on a scale from 1 to 5; (2) “Do sounds cause you pain or physical discomfort?” with the answers “yes, no, or I do not know.” We named these screening questions “loudness hyperacusis” and “pain hyperacusis” based on a recent review paper from worldwide hyperacusis experts [2]. They defined loudness hyperacusis as “…present when moderately intense sounds are judged to be very loud compared with what a person with normal hearing would perceive.” With respect to pain hyperacusis they state the following: “Some with hyperacusis experience pain at much lower sound levels than listeners with normal hearing.”

Statistical analyses were performed with SPSS 22 (SPSS Inc., USA). First, sample characteristics were calculated by using mean ± standard deviation and absolute and relative frequencies of hyperacusis for the sample. Second, we investigated the association of the hyperacusis parameters by correlating the GÜF sum score and the hyperacusis screening questions. Third, based on these analyses we aimed at defining two groups of tinnitus patients, with and without hyperacusis, and calculated a linear discriminant analysis using the GÜF sum score as the independent variable. Fourth, we calculated associations of the screening questions with the single items of the GÜF to investigate the association on a single item level. Complementary, we did a principal component analysis (PCA) with the principal axis factoring method using varimax rotation and three factors to validate the factor structure of the validation of GÜF. The PCA was done with and without the hyperacusis screening items, for the whole sample and for the subgroup with only hyperacusis to test for possible influences of sample and analysis bias. Fifth, we correlated the resulting factors with tinnitus- and non-tinnitus-related parameters to control for the external validity of these factors. Correlation analyses for metric variables were done with Pearson correlation coefficients, for categorical variables with chi-square test of independence, and for mixed metric and categorical variables by using Student's *t*-tests or analyses of variance (ANOVAs). For PCA, we report the highest factor loading per item.

## 3. Results

### 3.1. Sample Characteristics

Patients were 53.4 ± 12.1 years old, 65.8% male (106 of 161), and had a tinnitus duration of 117.4 ± 105.3 (*n* = 151) months and a tinnitus distress level of 43.7 ± 15.8 as indicated by the TQ and of 51.7 ± 23.6 as indicated by the THI. The BDI showed mild depressivity (8.3 ± 6.0) which was mirrored by diminished quality of life scores (physical health: 14.5 ± 3.2 (*n* = 159); psychological health: 13.9 ± 2.9 (*n* = 159); social relationships: 14.5 ± 3.6 (*n* = 160); environment: 16.4 ± 2.3 (*n* = 160)). Sixteen (9.9%) showed purely right, 34 (21.1%) purely left, and 111 (69%) tinnitus in both ears or within the head. Mean hearing level was 23.1 ± 15.5 dB HL (*n* = 148). Numeric ratings were in the upper half of the scale (loudness: 6.7 ± 2.2; discomfort: 7.3 ± 2.2 (*n* = 159); annoyance: 6.9 ± 2.4; ignorability: 7.0 ± 2.4; unpleasantness: 7.0 ± 2.3). The mean hyperacusis score as obtained by the GÜF was 15.4 ± 9.2. In comparison to the tinnitus sample of the validation paper [4] GÜF values were comparable (17.8 ± 9.2). For the pain hyperacusis screening question, 102 (63.4%) answered with yes and 59 (36.6%) with no. An additional 19 subjects rated the question with “I do not know” and were not considered in this analysis. Regarding the loudness hyperacusis question, 16 (9.9%) answered with never, 16 (9.9%) with rarely, 58 (36%) with sometimes, 27 (16.8%) with usually, and 44 (27.3%) with always.

### 3.2. Correlation Analyses of Hyperacusis Scores

**Figure 1:**
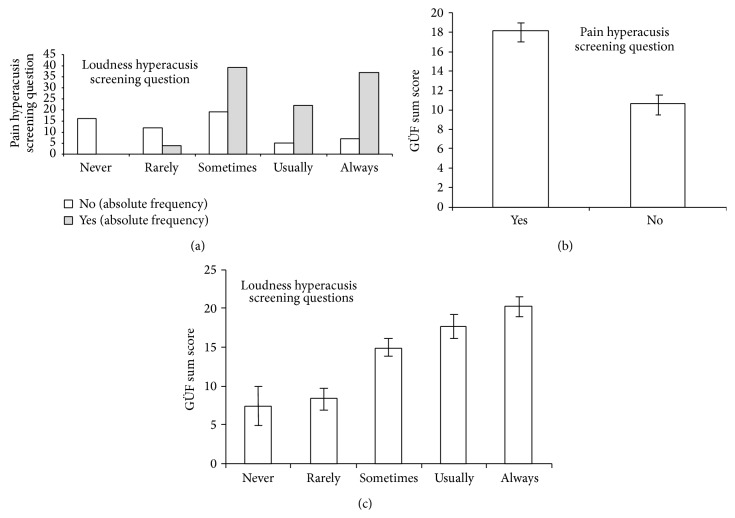
Association of pain and loudness hyperacusis screening question (a), of the GÜF (engl. hypersensitivity to sound questionnaire) sum score and pain hyperacusis screening question (b), and of the GÜF and the loudness hyperacusis screening question (c).

Association analyses are shown in Figure 1. The chi-square test showed a significant association of both screening questions (*χ* ^2^ = 50.147; df = 4; *p* < 0.001). The higher the prevalence of loudness hyperacusis (from never to always) the higher the frequency of patients reporting pain hyperacusis, with “never” and “rarely” showing almost no positive pain hyperacusis answers. Pain hyperacusis was associated with increased GÜF scores (yes: 18.2 ± 8.1; no: 10.7 ± 9.3; *T* = 5.385; df = 159; *p* < 0.001) and loudness hyperacusis was positively associated with the GÜF score (*F* = 11.089; df = 4,156; *p* < 0.001) with no differences between the answers “never” and “rarely” (*p* = 0.749), a significant difference between “rarely” and “sometimes” (*p* = 0.005), no difference between “sometimes” and “usually” (*p* = 0.149), and no difference between “usually” and “always” (*p* = 0.209). The answers “never” and “rarely” differed from the other three answers (all *p*-values < 0.005), and the answer “sometimes” differed from “always” (*p* = 0.001). To sum up, patients reporting pain hyperacusis and at least “sometimes” in the loudness hyperacusis screening question seem to suffer from hyperacusis as identified with the GÜF.

We defined groups of tinnitus patients with and without hyperacusis based on these values (hyperacusis: pain hyperacusis = “yes” and loudness hyperacusis ≥ “sometimes”) and calculated a linear discriminant analysis using the GÜF sum score as the independent variable. Wilks lambda was significant (*λ* = 0.821; *χ* ^2^ = 29.312; df = 1; *p* < 0.001) meaning that 82% of the total (within and between groups) variability could not be explained by the group difference. For all the cases, 70.8% were classified correctly with sensitivity of 64.3% and specificity of 81.0%. The cut-off was at 14.59 GÜF sum score. Student's *t*-test between groups was significant (no hyperacusis: 10.71 ± 18.47; hyperacusis: 18.47 ± 8.00; *T* = 5.683; df = 159; *p* < 0.001).

### 3.3. Correlation Analyses on Item Level

On the item level both screening items were significantly (on a Bonferroni level) associated with GÜF items 3, 5, 6, 10, 13, and 14 which are associated with hearing problems and fear-related emotion or behavior such as withdrawal. Hearing related items 8 and 3 were highly associated with the loudness hyperacusis screening question, and earache item 11 was especially associated with pain hyperacusis (see Table 1).

Principal component analyses (PCAs; Table 1) with all GÜF and the two hyperacusis screening items fulfilled the statistical requirements (Kaiser-Meyer-Olkin measure: KMO  =  0.901; Bartlett's test: *p* < 0.001). Factor one consisted of the GÜF items which were not associated with the screening items as indicated by *t*-contrasts and *F*-tests except for item 10 (anger about loud sounds). This factor represents problems or emotional distress related to reduced quality of daily life including family-related problems, ruined life, social withdrawal, and disturbed enjoyment of music amongst others. We name this factor “*quality of daily life*.” Factor two consists of GÜF items which represent fear and fear-related behavior such as avoidance behavior of sounds, but also one earache item and the two screening items for pain and loudness hyperacusis. However, the loudness hyperacusis question asked about loud and also hurtful sounds. Thus, we call this factor “*fear-pain hyperacusis component*.” The third factor is related to “*hearing difficulties*.” PCA without the screening items showed the same factor structure except for item 10 (anger about loud sounds) which then was added to the factor “*fear-pain hyperacusis*” (Kaiser-Meyer-Olkin measure: KMO = 0.904; Bartlett's test: *p* < 0.001). Data of this PCA are not shown in Table 1. PCA without the screening items and including only hyperacusis patients (as defined by the screening items; see above; see Table 1) also showed a comparable factor structure except for items 1 (fear of former not-disturbing sounds) and 15 (disturbed enjoyment of music) which was added to the factor “*hearing problems*” and except for item 10 (anger about loud sounds) which was added to “*fear-pain hyperacusis*” (Kaiser-Meyer-Olkin measure: KMO = 0.832; Bartlett's test: *p* < 0.001).

### 3.4. External Validation of the Generated Hyperacusis Factors

We extracted the three factors with regression analyses and correlated the individual factor loadings with nonhyperacusis parameters to test for external validity (Table 2). The first factor “*quality of life*” was specifically associated with THI, BDI, and quality of life scales, the numeric rating scale discomfort, and the scores of all TQ subscales except for the auditory subscale. The second factor “*fear-pain hyperacusis*” was associated with the numeric rating scales: annoyance, ignorability, and unpleasantness. Factor three “*hearing-related problems*” was associated with the numeric rating loudness, the mean hearing level, and the auditory subscore of the TQ.

## 4. Discussion

It was recently demonstrated in a large worldwide sample of tinnitus patients that the prevalence of hyperacusis in chronic tinnitus is about 55% [9]. Hyperacusis was defined by the screening question “Do sounds cause you pain or physical discomfort?” which corresponds to “*pain hyperacusis*” according to the recent classification by Tyler and colleagues [2]. In the present sample which only included patients from the Tinnitus Center Regensburg (Germany) a similar prevalence of 63% was found. We defined the parameter “*loudness hyperacusis*” based on the classification of Tyler et al. [2] as the answer to the question “Do you have a problem tolerating sounds because they often seem much too loud? That is, do you often find too loud or hurtful sounds which other people around you find quite comfortable?” In our sample 19.8% of the patients answered “never” or “rarely,” while 36% reported suffering from loudness hyperacusis “sometimes” and 44.1% “usually” or “always.” The mean GÜF sum score in our sample was 2.4 points lower than in the original validation sample [4]. However the validation sample [4] consisted only of tinnitus patients reporting hyperacusis. The mean score of both samples was in the medium impaired quartile of the GÜF (quartiles: light, medium, heavy, and very heavy). Prevalence of over 50% in our former and the present analysis and of about 40% in earlier studies [6] highlights a major role of hyperacusis in chronic tinnitus and the need for detailed assessment of this comorbid condition. This is particularly relevant because of the high need of therapy in this subgroup of patients with chronic tinnitus [9].

Since a detailed assessment of hyperacusis by specific questionnaires is not feasible in clinical routine, there is a need for validated screening questions. Based on this idea, we aimed at testing the suitability of the TSCHQ items for “*pain hyperacusis*” and “*loudness hyperacusis*” as screening questions for hyperacusis in chronic tinnitus. Indeed, both questions correlated significantly with the GÜF sum score, the here defined measure for hyperacusis. It turned out that the best cut-off for the five-point scale of loudness hyperacusis is between “rarely” and “sometimes” based on association of loudness hyperacusis with pain hyperacusis and the GÜF sum score (Figure 1). To conclude, both screening items might be helpful in screening tinnitus patients for hyperacusis.

On the other hand it turned out that most of variability of the group difference based on the GÜF sum score is not explained by only focusing on the screening items. After dividing the sample into groups with and without hyperacusis based on the two screening questions a discriminant analysis was calculated based on the GÜF sum score. With a cut-off of 14.59 GÜF scores Wilks *λ* and *t*-test between groups were significant; however 82% of variability is not explained at 71% correctly classified patients. This could be due to the multidimensional character of hyperacusis which cannot be fully identified with these measures. A further explanation may be that the screening items and the GÜF are measuring slightly different dimensions of hyperacusis.

The item-based analyses including *t*-tests, ANOVAs, and PCAs showed that both screening questions are associated with a factor which we defined as “*fear-pain hyperacusis*” which included items with fear-related and avoidance behavior content and pain-related items. One GÜF item regards earache, one screening item asks directly about pain, and one screening item asks about loud and hurtful sounds. Our findings confirm that the screening questions are mainly addressing fear- and pain-related mechanisms of hyperacusis in chronic tinnitus. It also implies that both screening questions addressing similar aspects of hyperacusis despite the different wording. Face validity is not fulfilled for these two questions, which might be related to the context of the data collection. The two hyperacusis questions are part of 35 questions about the tinnitus case history. Thus, even if these two questions are sensitive for the detection of hyperacusis and even if their wording refers to the two main aspects of hyperacusis (loudness and pain) they seem not to be sensitive enough to differentially detect the hyperacusis aspects “*hearing difficulties*” and “*quality of life*.” This view is also confirmed by the high intercorrelation of both screening questions.

Beside fear-pain hyperacusis, other hyperacusis-related dimensions in chronic tinnitus were found to be associated with quality of daily life and hearing problems. The factor structure showed high external validity as shown by correlations with tinnitus-specific and tinnitus-unspecific parameters. Quality of life was associated with tinnitus, depressivity, and quality of life questionnaires. Hearing problems were related to mean hearing loss, tinnitus loudness, and the auditory subscore of the TQ. Fear-pain hyperacusis was related to annoyance, ignorability, and unpleasantness of tinnitus. Two conclusions can be drawn. First, patients with tinnitus, hyperacusis, and hearing loss cannot assign their hearing difficulties and their impaired quality of life specifically to one of the three conditions. This has to be considered in the interpretation of questionnaire scores. In other words, if a patient scores high in hearing difficulties in the hyperacusis questionnaire, hearing loss and tinnitus have to be considered as relevant confounding factors. Second, different dimensions of hyperacusis have also been postulated. An expert consensus suggested that loudness, fear, annoyance, and pain comprise hyperacusis [2]. The postulated annoyance dimension which is defined as “negative emotional reaction to sounds” manifesting as “irritation, anxiety, and tension” might be closely related to our fear-pain factor. The fear dimension of hyperacusis was thought to reflect avoidance behavior. Our analysis suggests that annoyance and fear hyperacusis are highly associated and can be represented in one dimension. Typically, anxiety disorders are associated with avoidance behavior as stated in the classification systems DSM 5 and ICD-10 [16, 17]. The role of anxiety and avoidance behavior in hyperacusis was recently corroborated [3]. The initial validation of the GÜF revealed the factors “cognitive reactions to hyperacusis,” “actional/somatic behavior,” and “emotional reaction to external noises” [4]. The hyperacusis questionnaire (HQ) showed three factors: attentional, social, and emotional [5]. To sum up, although the multiple dimensions of hyperacusis are not yet well understood, there is clear evidence for the factors fear, pain, and loudness. Components such as hearing problems, quality of life, cognitive and emotional reactions, and behavioral responses should be the focus of future studies.

## 5. Conclusion

We could demonstrate that the screening items of the TSCHQ are suitable to screen patients with chronic tinnitus for hyperacusis and are particularly sensitive for the hyperacusis-related aspects fear and pain. Our analyses revealed quality of life and hearing as further important dimensions of hyperacusis. In the assessment of the impact of hyperacusis on quality of life and hearing, tinnitus and hearing loss have to be taken into account as confounding factors.

## Figures and Tables

**Table 1:** Association of hyperacusis screening and questionnaire items.

	Pain hyperacusis screening question *t*-test (df = 159)	Loudness hyperacusis screening question *F*-test (df = 4,156)	Factor 1 loadings Quality of daily life	Factor 2 loadings Fear-pain hyperacusis	Factor 3 loadings Hearing-related problems
GÜF items					

(1) Fear of former not-disturbing sounds	*t* = 2.854; *p* = 0.005	*F* = 3.240; *p* = 0.014	0.411		(0.307)
(2) Worries to habituate	*t* = 2.325; *p* = 0.021	*F* = 2.577; *p* = 0.040	0.592 (0.575)		
(3) Problems to listen in environmental noise	***t* = 5.042**; ***p* < 0.001**	***F* = 11.682**; ***p* < 0.001**			0.759 (0.857)
(4) Social tensions with family	*t* = 0.754; *p* = 0.452	*F* = 1.093; *p* = 0.362	0.547 (0.419)		
(5) Sound avoidance	***t* = 9.706**; ***p* < 0.001**	***F* = 16.703**; ***p* < 0.001**		0.797 (0.479)	
(6) Fear of noise	***t* = 4.510**; ***p* < 0.001**	***F* = 10.943**; ***p* < 0.001**		0.594 (0.520)	
(7) Ruined life due to hyperacusis	*t* = 1.257; *p* = 0.211	*F* = 2.261; *p* = 0.065	0.810 (0.853)		
(8) Hearing problems in environmental noise	*t* = 2.861; *p* = 0.005	***F* = 6.028**; ***p* < 0.001**			0.601 (0.653)
(9) Social withdrawal of others	*t* = 1.242; *p* = 0.216	*F* = 0.695; *p* = 0.597	0.572 (0.484)		
(10) Anger about loud sounds	***t* = 3.497**; ***p* = 0.001**	***F* = 5.613**; ***p* < 0.001**	0.508	(0.594)	
(11) Earache due to loud sounds	***t* = 4.475**; ***p* < 0.001**	*F* = 2.132; *p* = 0.079		0.445 (0.398)	
(12) Daily life problems if problems persist	*t* = 2.976; *p* = 0.040	*F* = 2.534; *p* = 0.042	0.727 (0.628)		
(13) Withdrawal from loud sounds	***t* = 5.274**; ***p* < 0.001**	***F* = 10.471**; ***p* < 0.001**		0.616 (0.670)	
(14) Fear that sounds are damaging	***t* = 3.588**; ***p* < 0.001**	***F* = 4.310**; ***p* = 0.00**2		0.533 (0.781)	
(15) Disturbed enjoyment of music	*t* = 2.146; *p* = 0.033	*F* = 2.453; *p* = 0.048	0.435		(0.335)

Screening questions					

Loudness hyperacusis	—	—		0.591	
Pain hyperacusis	—	—		0.653	

Bold font indicates significant effects corrected by Bonferroni (0.003 = 5%/15 contrasts per screening question). Numbers in brackets indicate factor loadings of patients with tinnitus and hyperacusis only.

**Table 2:** Association of hyperacusis factors with nonhyperacusis parameters.

	Factor 1 loadings Quality of daily life	Factor 2 loadings Fear-pain hyperacusis	Factor 3 loadings Hearing-related problems
Mean hearing level	*r* = 0.184; *p* = 0.025	*r* = −0.052; *p* = 0.534	***r* = 0.253**; ***p* = 0.002**
THI	***r* = 0.499**; ***p* < 0.001**	*r* = 0.310; *p* < 0.001	*r* = 0.101; *p* = 0.201
BDI	***r* = 0.426**; ***p* < 0.001**	*r* = 0.323; *p* < 0.001	*r* = 0.141; *p* = 0.098
Quality of life: physical health	***r* = −0.361**; ***p* > 0.001**	*r* = −0.263; *p* = 0.001	*r* = −0.206; *p* = 0.009
Quality of life: psychological health	***r* = −0.497**; ***p* < 0.001**	*r* = −0.312; *p* < 0.001	*r* = −0.159; *p* = 0.045
Quality of life: social relationships	***r* = −0.351**; ***p* < 0.001**	*r* = −0.241; *p* = 0.002	*r* = −0.147; *p* < 0.063
Quality of life: environment	***r* = −0.368**; ***p* < 0.001**	*r* = −0.195; *p* = 0.014	*r* = −0.162; *p* = 0.040
TQ total score	***r* = 0.543**; ***p* < 0.001**	*r* = 0.316; *p* < 0.001	*r* = 0.204; *p* = 0.010
TQ emotional subscore	***r* = 0.563**; ***p* < 0.001**	*r* = 0.340; *p* < 0.001	*r* = 0.088; *p* = 0.280
TQ cognitive subscore	***r* = 0.560**; ***p* < 0.001**	*r* = 0.275; *p* = 0.001	*r* = −0.089; *p* = 0.280
TQ intrusiveness subscore	***r* = 0.366**; ***p* < 0.001**	*r* = 0.287; *p* < 0.001	*r* = 0.211; *p* = 0.008
TQ auditory subscore	*r* = 0.258; *p* = 0.001	*r* = 0.327; *p* < 0.001	***r* = 0.553**; ***p* < 0.001**
TQ sleep subscore	***r* = 0.201**; ***p* = 0.011**	*r* = 0.052; *p* = 0.516	*r* = −0.119; *p* = 0.139
TQ somatic subscore	***r* = 0.264**; ***p* = 0.001**	*r* = 0.181; *p* = 0.025	*r* = 0.190; *p* = 0.018
Rating scale loudness	*r* = 0.106; *p* = 0.179	*r* = 0.161; *p* = 0.041	***r* = 0.202**; ***p* = 0.010**
Rating scale discomfort	***r* = 0.180**; ***p* = 0.023**	*r* = 0.159; *p* = 0.046	*r* = 0.016; *p* = 0.845
Rating scale annoyance	*r* = 0.107; *p* = 0.177	***r* = 0.232**; ***p* = 0.003**	*r* = 0.154; *p* = 0.051
Rating scale ignorability	*r* = 0.184; *p* = 0.020	***r* = 0.222**; ***p* = 0.005**	*r* = 0.169; *p* = 0.032
Rating scale unpleasantness	*r* = 0.102; *p* = 0.198	***r* = 0.222**; ***p* = 0.005**	*r* = 0.159; *p* = 0.044

Bold font indicates the highest association within each external parameter; please note that quality of life is inversely coded explaining the inverse correlation coefficients.
